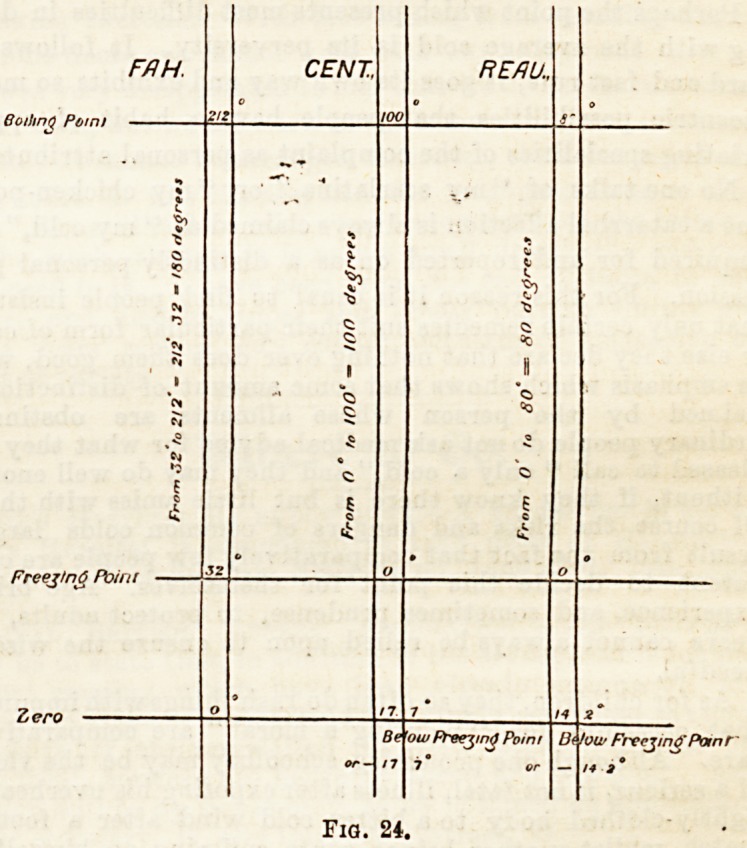# "The Hospital" Nursing Mirror

**Published:** 1896-06-20

**Authors:** 


*The Hospital*, June 20, 1896. *Extra Supplement.*

### "The Hospital" Nursing Mirror.

Being the Extra Nursing Supplement of "The Hospital" Newspaper.

[Contributions for this Supplement should be addressed to the Editor, The Hospital, 428, Strand, London, W.C., and should have the word "Nursing" plainly written in left-hand top corner of the envelope.]

### News from the Nursing World.

### THE ROYAL HOSPITAL, RICHMOND.

H.R.H. the Duchess of York has fixed July 8th for the opening of the new children's ward at the Richmond Royal Hospital, which is to be called after her the "Princess May" Ward. The Duchess will receive purses in aid of the furnishing fund after the ceremony.

### NORTH-EASTERN HOSPITAL FOR CHILDREN.

A purse of £1,000 will, it is hoped, be presented to the Duchess of Connaught at the Rose Fête which takes place next week in aid of this hospital. The contributions started with a donation of £100 from the Duke of Westminster, and a similar sum has been given by Lord Amhurst of Hackney.

### THE GREAT NORTHERN CENTRAL HOSPITAL.

A large audience gathered in the Queen's Hall on June 11th, when a concert, under the direction of Sir Augustus Harris, was given in aid of the Ladies' Association Endowment Fund of the Great Northern Central Hospital. The Duchess of York founded the endowment fund for the maintenance of the "Victoria Mary" Ward, and takes a keen interest in its welfare. Purses were presented to her Royal Highness before the concert began, amongst which were several containing handsome contributions. The Prince and Princess of Wales and their daughters, with the Duke of York, who is the president of the hospital, were also present.

### A "QUIET DAY" FOR HOSPITAL WORKERS.

We are asked to state that arrangements have been made to hold a devotional "Day of Rest" at Hertingfordbury, Hertford, on Thursday, June 25th, for the benefit of hospital chaplains and nurses, Bishop Thornton having undertaken to give the addresses. The return railway fare from King's Cross to Hertingfordbury will be 2s. 6d., and will be the sole expense to those accepting the invitation. All hospitality will be voluntarily offered to visitors. The church is close to the station, and further particulars may be obtained from Canon Burnside, at the Rectory. Nurses wishing to be present are requested to communicate with the Rev. C. H. Bowden, Chaplain's House, Guy's Hospital, S.E., at once.

### CHILDREN'S HOSPITAL, PADDINGTON GREEN.

The wards of the new hospital on Paddington Green are quite ideal. The bright, clean effect of the terrazzo floor, tiled walls, with nursery rhymes artistically portrayed thereon, and Turkey-red screens and cot covers, is most pleasing, and a closer inspection reveals the care with which all dust-traps are avoided in this modern children's hospital. All corners are rounded, inside blinds and lockers are forbidden, and everywhere everything is spotless. The balconies overlooking the Green are pleasant places for small convalescents to sit in. It is a pity that the space "behind the scenes"—bath-rooms, offices, etc.—are cramped, even to the extent of there being only one larder for milk and meat alike for the whole hospital. The nurses' quarters, too, want a little attention. The one small sitting-room, intended to serve also as dining-room, sadly needs a few pictures and books and easy chairs to make it a home-like apartment. Surely some friends of the hospital might make it their business to see not only that the little patients have all they require, but that those who tend them are supplied with the simple comforts which are their due.

### AT ST. THOMAS'S HOSPITAL.

Hitherto the nursing staff of St. Thomas's Hospital have had no specially reserved corner of the grounds for their own particular use. Just lately it has been proposed that the central quadrangle, facing the counting-house, shall be set apart for this purpose, where, as the writer in the "St. Thomas's Hospital Gazette" feelingly puts it, the nurses may be "free from patients, drugs, students, and everything connected with their daily routine work." Doubtless the nurses will much appreciate this arrangement for their comfort, and in this hot weather will find their own quarangle the most restful place in which to spend off-duty times.

### NEW HOSPITAL FOR WOMEN.

A pleasant little social gathering took place last Friday afternoon at the New Hospital for Women, Euston Road, when the matron and medical staff were "at home." The guests found tea ready for them in the lecture room on the ground floor, and afterwards wandered at will over the building. The wards were most artistically flower-decked, mostly with graceful Iceland poppies and grasses fetched at an early morning hour from Covent Garden by the nurses. The wards are charming at all times, so bright and cheerful, and were in holiday trim on this occasion with new "bed-spreads" of a dark blue and white linen. Every corner of the hospital was inspected with interest by the visitors, from the out-patient department to the kitchen on the top storey, and the nurses and servants' quarters.

### MEDICAL WOMEN IN AUSTRALIA.

Numbers of women have been following the medical course at the University of Melbourne ever since it was open to them, now about eight years ago. So far they have all been natives of Melbourne, and have settled down to practise in that town and its suburbs. The first to go further afield is Dr. Grace Vale, who graduated in 1894. She has recently settled in Ballarat, the second largest town in Victoria, and is there reported to be doing very well.

### "COTTAGE NURSES."

The annual conference of the Affiliated Benefit Nursing Associations was held at Stafford House, by permission of the Duke and Duchess of Sutherland, in the first week of June. Mr. J. J. Bickersteth presided, and gave some particulars of the branch association in Yorkshire, which he represented. Miss Broadwood, the originator of the parent scheme, described its objects and working, and the Duchess of Sutherland gave the conference details of the Sutherland Association. We hope to give Miss Broadwood's address in full next week, dealing, as it does, with a problem which is increasingly forcing itself upon the public attention.

### NURSING ARRANGEMENTS AT CARDIFF INFIRMARY.

The General Committee of the Cardiff Infirmary have provisionally adopted for six months (beginning on July 1st next) a scheme whereby they hope to encourage nurses to remain on the staff for a longer period than is found to be at present the case. It is proposed "that nurses during the second year should receive a salary of £16, for the third year £20, for the fourth £25, for the fifth £25, and 10 per cent. on the amount received for private nursing; for the sixth year £28, and 15 per cent. on the amount received for private nursing; the seventh year £30, and 15 per cent. on the amount received for private nursing." The gross earnings of the nurses for 1893 were shown to be £1,060 1s. 1d., profits £573 19s. 8d.; for 1894 the gross earnings were £841 11s. 9d., profits £333 13s. 8d.; for 1895 the gross earnings were £696 7s. 4d., profits £200 12s. 2d. On the suggestion of Dr. Wallace, it was decided "that nurses in the fourth year, if sent out private nursing, should receive 5 per cent. of their earnings in addition to their salary." It would appear from the words "*if* sent out private nursing" that the hospital and private nurses are interchangeable. This is a custom which has been proved to be undesirable, and has been abandoned at the best managed institutions. The managers of the Cardiff Infirmary would do well, while they are making alterations, to arrange to keep the private nursing institution and the infirmary nursing of the infirmary patients distinct and separate.

### NURSES IN NEW ZEALAND.

Hospital nurses in Auckland seem to have pretty hard times, judging from a letter from a medical man which recently appeared in the *New Zealand Herald.* The trained nurses in the Auckland Hospital, "educated women of a superior degree of intelligence," are "actually required to clean windows, to wash drawsheets . . . and do various works of the scrubbing kind." "The long hours," continues Dr. Bakewell, "are contrary to all the principles enunciated by Miss Nightingale in her treatise on nursing. And the folly of sending a night nurse to do eleven hours' work on bread and butter and tea can hardly be surpassed." Clearly reforms are urgently needed in Auckland.

### TRAINED NURSES' CLUB.

We are asked to state that a lecture will be given on Friday evening, June 26th, at 7.45, at the Midwives Institute and Trained Nurses' Club, 12, Buckingham Street, Strand, by Dr. Robert Lee. The subject will be "Maternal Impressions." Tickets can be obtained by non-members for 6d. each.

### WHAT IT IS TO BE A LADY!

From the list of medical vacancies in the *British Medical Journal* we cull the following which, in the distinction suggested, is rather neat: "Medical Woman as Clinical Assistant for the Out-patient Department; and Lady as Assistant Dispenser."

### LADY WIMBORNE AT POOLE.

A sale of work was lately opened by Lady Wimborne in the grounds of the Cornelia Hospital at Poole. The proceeds will go to the support of the "Poole Cot" which is maintained at Dr. Barnardo's "Babies' Castle."

### A DISTINGUISHED WOMAN.

A medical woman, Dr. Winifred Dixon, has lately been appointed by the Irish Royal College of Surgeons examiner in midwifery and gynæcology. Miss Dixon is assistant physician to the Coombe Lying-in Hospital, Dublin, and a member of the staff of the Richmond (House of Industry) Hospital. She won for herself considerable distinction during her student career, and is a Fellow of the Royal College of Surgeons, holding also the degree of Doctor of Medicine in the Royal University of Ireland.

### PRIVATE NURSING.

A correspondent raises a question of payment upon which it would be interesting to have the opinion of private nurses working on their own account. She says: "I have to be thankful for the success I have had. . . . I think it is due to taking any case that really needs nursing, and where patients' circumstances are poor I have asked them what they could really afford and taken it. I know many trained nurses will disagree with me on this point and say that doctors will not help a nurse to get her full fee if she once takes less. But I have not found it so. I sometimes nurse a poor man who cannot afford more than 10s. 6d. a week, and perhaps my next case will be one at which I can get three guineas, possibly both attended by the same medical man. He does not charge the poor man on the same scale as his wealthy patients, why should the nurse?"

### THE LADIES' CHARITY DISPUTE AT LIVERPOOL.

We are glad to note that among the subscribers to the Ladies' Charity at Liverpool, many are still found who have not bowed the knee to that Baal—the board of management. We understand that now that arbitration has been refused, a majority of the ladies' committee desire to make it publicly known that they do not support the action of the board of management in the present dispute with the late medical staff. As we have already pointed out, it is essential, both for the safe working of the charity and for the proper training of the midwives, that the hospital should have a properly qualified staff, on whom should be placed complete responsibility for the proper treatment of the patients.

### SHORT ITEMS.

A bazaar for the benefit of the nursing staff of the Trinity College Mission (St. George's, Camberwell) was opened last week at the Mission Hall by Lady Amherst of Hackney.—Lady Rose Weigall has been elected on the Isle of Thanet Board of Guardians. The contest was fought on the question of compulsory vaccination, of which Lady Rose is a staunch supporter.—The fancy fair lately held at Shortlands in aid of Guy's Hospital Re-endowment Fund has resulted in a sum of over £1,000 being handed over to the hospital.—A garden party will be held in the grounds of the British Home for Incurables, Streatham, on the afternoon of Tuesday, June 30th, at which the Duchess of Sutherland has kindly consented to open a sale of the inmates' work for their own benefit. —The Countess of Winchilsea was present at an "At Home" held at the Maternity Charity and District Nurses' Home, Plaistow, E., on Saturday last.—It is proposed to start an association to provide trained district nurses for Thrapston, Sudborough, Islip, and surrounding parishes. A meeting to consider the scheme was held at Islip Rectory last month.—The new headquarters of the Volunteer Medical Staff Corps, Calthorpe Street, Gray's Inn Road, were opened by Princess Louise, Marchioness of Lorne, on Wednesday, June 10th.—The Princess of Wales intends visiting the wards of St. Mary's Hospital one day during the present week.

### Hygiene: For Nurses.

By John Glaister, M.D., F.F.P.S.G., D.P.H.Camb., Professor of Forensic Medicine and Public Health, St. Mungo's College, Glasgow, &c.

### XI.—MODES OF HEAT MEASUREMENT—THERMOMETERS—TEMPERATURE SCALES.

As many diseases which come under the care of a nurse exhibit febrile disturbance, and as it is necessary for the information of the physician that the daily periodic temperatures should be recorded, it is of the utmost importance that the nurse should be familiar with the instrument by which such temperatures are registered. There are two ways of estimating the temperature of a patient, one of which is entirely wrong and fallacious, and the other scientific and correct. Before the thermometer came to be so much used, the habit was to judge of the patient's condition by the apparent relative difference of temperature of the hand of the observer and the body of the patient. This was but a rough-and-ready method, and has fallen, very properly, into desuetude. The modern method is by the thermometer, Fig. 23, which is the heat-measurer. The clinical thermometer is probably the principal tool of the nurse. She ought, therefore, to know not only how to use it, but its merits also, and how it is made. What is a thermometer? It consists of a glass tube of uniform capillary bore terminating at one end in a bulb. Part of the tube, and the whole of the bulb, contains mercury; the other end of the tube being sealed. The space between the upper level of mercury and the sealed end is a partial vacuum. A scale, divided into equal parts, called degrees, and into sub-divisions of a degree, either engraved on the glass of the tube, or on paper enclosed within an outer glass tube, or on the wooden framework of the instrument, is an essential part of the thermometer. Clinical thermometers usually have the scale engraved on the glass, thermometers for chemical purposes on paper, and ordinary thermometers for varied use on a wood or metal frame. The temperature-range of the instrument varies, depending upon the purpose for which it is to be used. For example, the range of a clinical thermometer is usually from 92° to 112° Fahr., these extremes being beyond the temperatures, either below or above, which are compatible with life; the chemical thermometer may either range between 0° and 100° Cent., or 0° and 360° Cent., or even higher, depending upon its destined use.

Fig. 23.

The glass portion of the thermometer having been made, it becomes necessary to "calibrate" the instrument as a heat measurer. Calibration means the graduation of the instrument in reference to the uniformity of its bore. Mercury, which it contains, like other liquid substances, expands definitely in proportion to definite amounts of heat, and it is chiefly used for the clinical thermometer because it enables a compact instrument to be made. Alcohol, coloured, is used by preference, however, for thermometers which are used in some other fields of observation. Graduation of the thermometer is a very important matter. In making an ordinary instrument the workman has two fixed points from which to operate, viz., the freezing point, and the boiling point of water. To get the former he places the bulb of the ungraduated instrument in melting ice, and when the mercury remains stationary he marks that level on the glass. To obtain the latter he suspends the bulb in the steam of boiling water, and when the mercury rises to its highest point he makes a second mark. If the glass tube of the instrument be of equal bore between these two points, he may "graduate" the stem by putting equidistant marks upon it. The number of degrees or "grades" (Latin, *gradus*—a step) on a scale may be of any number, according to the choice of the user, so long as they have equal and known value. In effect, however, the instruments now in use in the world are graded according to one or other of three different scales, and are called respectively the thermometers of Fahrenheit, Celsius or Centigrade, and Réaumur. The first is in common use in our own country; the second in most Continental countries, and for all scientific work; and the last mainly in Russia. Fahrenheit—a native of Dantzig—was the first, in 1720, to use mercury in thermometers; before that time alcohol was the registering fluid. He took as the limit of coldness the temperature of Dantzig in the winter of 1709, which was the same as that produced when the mercury was plunged into a mixture of snow and ammonium chloride, and he called that point zero, or freezing point. But in so doing he made a considerable mistake, whereby much confusion in the student mind has since resulted, for the point which he called zero was 32° below the actual freezing-point of water; therefore, freezing-point on his instrument is not synonymous with the zero of the other scales, but is 2° above it.

Celsius—a Swede—twenty-one years after, rectified this mistake by graduating his instrument from the melting-point of ice, which he called zero, and which has been the standard to this day. Between zero and boiling-point he made 100°, therefore his instrument and scale has come to be

Fig. 24.

called Centigrade (Latin, *centum*, one hundred; *gradus*, a step or grade).

Réaumur—a Frenchman—based the zero of his instrument on the same fact. Fahrenheit divided his scale from his supposed zero to boiling-point into 212, Celsius into 100, and Réaumur into 80°, or grades. The following diagrammatic figure will assist the student. Fig. 24.

The relative value of one degree of one instrument to one degree of the others ought to be known. In view of the possible establishment in this country of the metric system, and for other reasons, a nurse should be able to interpret these values. The first point to remember is that the freezing and boiling points of water at the sea-level are fixed. If all instruments had been so graduated, no difficulty would have emerged, and the determination of the relative ratios of the different scales would have been simple. Suppose, for example, the number of degrees in the Fahrenheit scale to have been 212° – 32° = 180°, in the Centigrade 100°, and in Réaumur 80°, the ratios would have been as follows, viz., 180 : 100 : 80, or ÷ 20 = 9 : 5 : 4; in other words, 9° Fahr. would equal 5° Cent. and 4° Réau., and conversely. But the freezing-point of each scale is, unfortunately, not the same; in Fahr. it is 32°, in Cent. 0°, and in Réaumur 0°. Since we work with the entire scale of each instrument, however, the factor of difference between the zero and freezing-point of the Fahr. scale must always be reckoned with.

The following table shows how to transpose correctly the scale of one instrument to that of the others.

1. To convert degrees Fahr. into degrees Cent. (subtract 32°, then multiply by 5 and divide by 9).

2. Fahr.° into Réaumur°, subtract 32, multiply by 4, and divide by 9.

3. Cent.° into R.° – Multiply by 4 and divide by 5.

4. Cent.° into Fahr.° – multiply by 9, divide by 5, add 32.

5. Réau.° into Fahr.° – multiply by 9, divide by 4, add 32.

6. Réau.° into Cent.° – multiply by 5, and divide by 4.

### Trained Nurses' Clinic.

### VII.—COMMON COLDS AND OTHER MATTERS.

How many serious illnesses are shown to have originated in an apparently trivial and ordinary cold! In certain climates the latter is looked upon as an almost inevitable accompaniment of the winter season, and hence often gets totally inadequate treatment. Natural as it seems to neglect such a common ailment, it is unwise to do so, and such a procedure often brings about most disastrous results.

Sometimes a mild winter conveys immunity from the customary cold, but during the spring that follows many who have boasted of their unusual freedom from catarrh are unexpectedly invalided by some form of the affection. "It's provoking to get a cold now, after escaping so well all the winter," says one who has been tempted perchance into indiscretion by the lightness and brightness of a chilly May day. Such a speaker often implies that circumstances and not personal carelessness are to blame for the calamity.

Hence the neglected cold is at the root of many a severe bronchial and gastric affection, as well as the attacks of pneumonia, rheumatism, &c., which make a sudden onslaught alike upon the weakly and the strong.

Perhaps the point which presents most difficulties in dealing with the average cold is its perversity. It follows no hard and fast rule, it goes its own way and exhibits so many eccentric possibilities that people have a habit of appropriating specialities of the complaint as personal attributes.

No one talks of "my scarlatina," or "my chicken-pox," but a catarrhal affection is always claimed as "my cold," and enquired for and reported on as a distinctly personal possession. For this reason it is usual to find people insisting that only certain remedies suit their particular form of cold, or else they declare that nothing ever does them good, with an emphasis which shows that some amount of distinction is claimed by the person whose ailments are obstinate. Ordinary people do not ask medical advice for what they are pleased to call "only a cold," and they may do well enough without, if they know there is but little amiss with them. Of course the risks and dangers of common colds largely result from the fact that comparatively few people are competent to decide this point for themselves. Age brings experience, and sometimes prudence, to protect adults, but years cannot always be relied upon to ensure the wisdom needful.

As for children, they so often do rash things with impunity, that occasions for "pointing a moral" are comparatively rare. Although one promising schoolboy may be the victim of a serious, if not fatal, illness after exposing his overheated, lightly-clothed body to a bitter cold wind after a football match, whilst another brings acute suffering on himself by lying on wet grass, hundreds of lads seem to take no harm from similar indiscretions.

No one grudges the healthy English schoolboy his immunity from the evils to which he rashly exposes his muscular little frame, though we sometimes wish that he knew the priceless value of the health thus imperilled.

Modern innovations in the way of clothing are perhaps the most practical precautions against taking cold that can be recommended. Wool in every form has come steadily into favour, and materials have gradually improved, and so have the forms of garments.

These outward and visible proofs of sanitary progress need still to be supplemented by prompt personal attention to the early symptoms of every form of cold. The doctor's aid is too often left unsought until grave symptoms develop; he finds to his chagrin that he is summoned too late for the preventive measures, which his skill could have indicated had the chance been offered at an earlier stage.

A nurse can do most valuable service to both patient and doctor in the matter of colds. It is her place to watch and wait, not to diagnose. It is for her to show all those with whom she is brought into contact how much can be done by intelligence and skill to ward off illness, and in all such preventive measures she will be sure of the approbation of medical men. So much nonsense has been talked about the trained nurse being the doctor's "assistant," that contempt has been justly aroused by such pretentiousness; but a sensible woman who has been wisely and thoroughly trained does not claim anything beyond the honourable and modest position which is rightly her own. A moment's reflection on the relative degrees of education provided severally for student and nurse shows how different is the future work expected from each. In three years' service in the wards women have opportunities for observing and tending the sick, of which the student in days to come may justly feel envious—but the five years' study and the position eventually filled by the qualified medical man or woman confers on them authority which no mere nurse, good and valuable as she is, can aspire to hold.

Therefore even the common cold should not be lightly diagnosed by patient or nurse—firstly, on account of its immediate symptoms which may be misleading, and secondly, because even a persistent cold shows a necessity for medical advice as to general health. Warm baths, rest in bed, light, nourishing food doubtless form good homely treatment, and the mother of a large family adds to these many remedies which experience has in the course of years brought practically to her knowledge. A continuous ailment, even if "only a cold," is depressing and weakening, and after effects can be warded off by the timely tonics or other drugs, the use of which medical intervention alone can justify. Amateur drugging is quite a passion with some people, and it needs all the tact a nurse has at command to discourage the practice without discouraging the person. New remedies, and such as bring down temperatures are as popular as they are dangerous in ignorant hands, and their use or exhibition by the trained nurse without a doctor's permission is an unpardonable breach of duty. It is her privilege to aid all with whom she comes in contact to preserve and regain their health, but she must suggest that medical advice be sought when even so ordinary an ailment as the common cold proves unamenable to homely remedies, and to do this and to serve the doctor faithfully and promptly should suffice to occupy the most fully trained of modern nurses.

### The Indian Nursing Service.

FROM A CORRESPONDENT.

As people in England seem to know very little about nursing in India, and almost nothing about military nursing in India, I trust you will publish these few words of mine in order that nurses wishing to join the Indian Nursing Service may know more about it than I did when I joined; and may be helped to avoid the mistakes which many members of our corps have made, and which have afterwards caused a good deal of inconvenience. So few sisters know what they are coming to when they join the Indian Nursing Service. Many say they would never have joined had they known this, that, and the other (I believe this is one form of homesickness); others would, probably, be glad to join us if they knew what a comfortable life we have out here, compared with our harder worked sisters in Europe.

We need not be pitied on the score of climate, for coming to work in a hot climate one must expect a certain amount of heat, and its attendant miseries, insects, thirst, prickly heat, and so on, and must make up one's mind to bear it. In one respect only are we less fortunate than our harder-worked sisters in Europe—we have far less independence and real freedom. Compelled by regulation to wear a uniform, which, although exceedingly pretty, is inconveniently conspicuous, we can be spotted at once among ever so great a crowd of people, and this uniform must be worn on or off duty, all day, indoors and out, whether visiting friends, attending garden parties, gymkhanas, or any other social function, and may only be left off when one is going out of an evening, on which occasions we have lately been allowed to wear ordinary ladies' evening toilet. This privilege was only granted this last year, and is much appreciated. We are not allowed to go to dances, nor to remain out very late, *i.e.*, past midnight, at any entertainment, and it is felt a great restriction, lately enforced, and I suppose rendered necessary by the over-indulgence of some indiscreet member whose folly has caused the making of a rule which binds us all, and prohibits an enjoyment which could not harm us, if indulged in with moderation. However, the pace must be set for the weakest horse in the troop, and one should not grumble.

When first I joined I was sent to a big station in the N.W.P., where there were four of us. We lived in a very old, cool bungalow, not quarters, as they were not then built. We had not our correct allowance of rooms, but we were quite happy without, as we got on well together, which was wonderful considering the different lives we had led and the different places we had come from. The senior sister, who did the housekeeping, was an Edinburgh nurse, and of our three selves, one was Birmingham trained, one London, and one Dublin. We did night work week and week about, the senior not doing it, as she had all the housekeeping, which in India is a serious undertaking. We breakfasted together at eleven, had afternoon tea in the verandah at quarter-past four, after which the sister on duty returned to her wards and the others went out for a ride or a drive until dinner, at eight, soon after which we turned in, as it is a case of early rising here. In winter we go on duty at seven a.m., and in summer at six or half-past six a.m., according to the amount of work to be done. In the hot weather we slept out in the compound or in the verandah under a punkah, the sway of which is calculated to make anyone sleep. We had a big ward of fifty beds, almost all cases of enteric fever, and three small rooms, generally inhabited by sick officers and their attendant orderlies, who act as nurses under us. Also we had another small room, where we kept our report-books, medicines, dressings, &c., which was called the duty-room, and where we sat when we had time, which was but rarely. Sisters are not supposed to be in their wards unless they are actually engaged in some way, and we are always allowed one or two orderlies to work under us, and they do all the rougher and heavier work, which saves our time and also our strength. Of course, they are not in any way trained nurses, but they make beds, wash patients, and are a great help to us. We lived close to the hospital, and used to walk to and fro in the evenings, and by day we had a large covered wagon, drawn by oxen, to take us up and down. It seemed odd at first jolting along in such a huge clumsy machine. When there were four of us in the station the work was not very heavy; but as each sister is allowed two months' leave during the year, we were necessarily working three only for eight months, and when one was called away to another station it made it heavier. When there were four of us working, one did night duty from nine p.m. till six a.m., one from six till breakfast, one breakfast till tea, the other from tea till dinner, and we changed on to night duty every week. When we were three only, one did the night, one six till two, the other two till eight. The senior sister arranges the hours on and off duty, she also does the house-keeping, mess accounts, and official correspondence, which is a heavy duty in a big station. There are four circles, each governed by a lady superintendent, and each station under her is managed by a deputy superintendent, and worked more or less differently according to the number of sisters stationed there, and the kind of cases prevailing. The general thing is enteric, ague, dysentery, and liver—very little surgical work at all. All over India we wear the same pretty dress, white cotton, with scarlet cuffs, collar, and belt, and white sailor hats (or, for church, dainty grey bonnets). In the cold weather we wear grey, with red facings. The only fault in the dress is that the stiff red collars (mine is made of tin and covered) are so hot, and the red buttons won't wash, and it is such a nuisance putting them in fresh every day; also one needs such an immense stock of white things—at least sixteen dresses, as we need one a day, and a corresponding number of cuffs and collars, as the perspiration soon renders them useless and unsightly. One of the first things I did was to learn to ride from a good riding-master, and I should advise all sisters to do the same, unless they can ride well before coming out. One can always keep a pony, and it is a pleasant way of taking exercise. With feet aching from standing about on stone floors one cannot walk with any enjoyment.

I was very disappointed to find that we do not have a ward of our own, but we all work in the same wards turn and turn about. It cannot be arranged otherwise, as no one could do long hours in this climate, and the arrangement is a wise one. But still it does take away somewhat from the interest in the work, though it is only a small drawback, and one gets used to it. I have been from one end of India to the other, and also in Upper and Lower Burmah during my three years' service, so I cannot complain of stagnation or want of variety.

### Where to Go.

GUY'S HOSPITAL RE-ENDOWMENT.—A garden *fête* in aid of Guy's Hospital is announced to take place at Hanworth, Middlesex (near Feltham Station) on June 23rd, 24th, and 25th.

ROYAL BRITISH NURSES' ASSOCIATION.—The secretary asks us to state that, in addition to the arrangements for the annual meeting which have been already announced, his Grace the Duke of Sutherland has kindly consented to allow members of the association to visit Stafford House on July 22nd, between the hours of three and five p.m. Admission will be by ticket only, to be obtained on application to the secretary, 17, Old Cavendish Street, W.

MIDDLESEX HOSPITAL.—The Duke and Duchess of York have consented to inaugurate the grand fête which is to be held in the grounds of the Middlesex Hospital on July 1 to celebrate the opening of the new convalescent home in connection with the hospital at Clacton-on-Sea. Tickets may be obtained from the secretary at the hospital. Prices, for the afternoon, one guinea each; for the evening, half a guinea each, or three for one guinea.

### Holidays and Health.

[Readers of The Hospital in need of information about health resorts at home or abroad, or desirous of aid in forming holiday plans, are invited to send queries to Editor, 428, Strand, W.C. (marked "Travel" on outside of envelope), which will be answered under this section.]

### THE FOREST OF DEAN.

There is probably no district in England which, at any rate till the last few years, has been so little known to outsiders as that of the Forest of Dean. Familiar as were its outskirts, abutting on the River Wye, to tourists travelling by road and river from Ross to Monmouth, the beautiful interior of the forest was an unknown land, save to the miners crossing its grassy paths to and from their work in the iron and coal mines. Something was done to bring the beauties of the forest to the knowledge of the traveller by the making of a passenger railway from Lydney, on the Severn, through the heart of the forest valley to Lydbrook on the Wye, and still further by the connection with the other side of the river by the Severn Bridge between Sharpness, near Berkeley on the Cotswolds, and Gatcombe or Purton on the Lydney or forest side. Even this did little, however, to open up the district, the line being from local circumstances a comparative failure, with a poor service of trains; and though well adapted for mineral traffic, it tapped the forest too far from Gloucester, which from its antiquity, picturesqueness, and convenient situation on the Great Western and Midland main lines, is the best starting point for travellers from north, south, east, and west. Further, at least a few years ago, the hotel accommodation was both bad and expensive, with the exception of the one really forest hotel, the Speech House. It has been a matter of wonder to lovers of the forest that no inducement has been offered by the Office of Woods, the custodians and managers on behalf of the people of the empire, for the erection of at least another hostel in some of the prettier parts of the forest itself, for it must be remembered that the idea of this being "Crown property" in the meaning of its belonging to the Sovereign is altogether a fiction, devised and fomented by local officers as a reason for non-progress in the matter of improvements; it cannot be too well grasped by the public that the Forest of Dean is not a close Royal borough, but belongs to the people, and they are entitled to the use of it just as much as they are to the use of the parks in London, neither more nor less.

But at least there is the quaint old Speech House, and if there is no place more restful to body, eye, and mind than the heart of the Forest of Dean, there is certainly no spot within its borders where seekers after quiet may better find what they want than among the glades which encircle on all sides this pleasant old hotel. From sitting-room and bedroom may be seen mile after mile of massed fir, oak, and beech, interspersed with the darker green of holly bushes, centuries old, grassy glades, and broad verdant roads, in places smooth and velvety, in other breast high in bracken; a glorious place, so peaceful, so restful, and withal full of the song of birds and the murmurous hum of the bee.

Within a walk, through acres of woodland, there are glimpses to be had of the Severn Sea, and the Cotswold Hills far in the distance; in another direction, with the help of a carriage supplied by mine host, or by train (Speech House Road Station is quite near) are the Highmeadow Woods, running down to the Wye, "swiftly flowing," with Symon's Yat, the "River Gate," surely one of the most charming spots in England. Or, again, making an early start, a pleasant excursion can be made by train to Ross, thence chartering a boat to Chepstow, past ancient Monmouth and its historic castle, past Tintern with its famous Abbey, and Chepstow Castle, also well worth a visit, returning home viâ Lydney and Speech House Road Station to supper and well-earned sleep. For this excursion a well-furnished luncheon basket is essential, with a good margin for the boatmen, who not only take you down with the stream, but bring the boat back against it. To thoroughly enjoy this expedition two or even three days should be allowed, halting one night at Monmouth, and then going on next day to Chepstow, so as to give plenty of time for Monmouth Castle, Tintern Abbey, the Wyndcliffe (from whence there is a good panorama of wood and river), and Chepstow Castle.

Another pleasant expedition can be easily made to English Bicknor, walking through the forest, and on by wooded paths overhanging the Wye, to Symon's Yat, already mentioned, and home in the cool evening to the friendly hotel. A day should be given to an equally pleasant sylvan walk to Danby Beeches; here from the keeper's lodge, hard by, can be procured the necessary kettle of hot water to make tea. The beauties of such a walk are never ending. Avenues of firs there are, miles in length, crossed and re-crossed with diverging grass drives and paths, fragrant and sweet with the luxurious smell of pine trees and bracken. In July the forest is brilliant with foxgloves, growing many feet high, and the mossy banks are rich with ferns of various kinds. In some places the pretty little oak fern grows abundantly; the beech fern, too, may be found more rarely. Enterprising explorers should be provided with a pocket compass, for those who dare to strike through the woods may chance to find themselves in a labyrinthine maze. It is well, therefore, to know the points of the compass, and where the direction of "home" lies, when it is not possible to go far wrong. Failing this precaution it is useful to remember that the lichen and mosses grow always on the north side of the trees, an observation in which the writer has often found salvation.

There is a charming walk through the heart of the forest to "The Bailey," overlooking Newnham and the Severn. It is far better to boldly strike the forest track rather than follow the road. Any little anxiety as to the path is well repaid by the infinitely greater beauty and interest of the way. To reach the forest the most direct way is, of course, viâ Gloucester, but as that is rather a shunned part of the world just now, it would be equally well to go to Bristol by the Great Western Railway, and thence through the Severn Tunnel to Port Skewet and Lydney, thus avoiding Gloucester and its plague-stricken environs. Intending visitors should write to Mr. Boyce, at the Speech House Hotel, near Coleford, Gloucestershire, who will be delighted to welcome holiday-making nurses, and writes to us that he will allow them 15 per cent. on all charges.

### Appointments.

St. Mark's Hospital for Fistula, City Road, N.—Mrs. Alice M. F. Hepper has been appointed Matron at this hospital. Mrs. Hepper was trained at the West London Hospital, afterwards holding appointments at the Cumberland Infirmary and the General Hospital, Wolverhampton. Since July, 1894, she has acted as assistant matron at the Hospital Convalescent Home, Swanley. We congratulate Mrs. Hepper on her promotion.

### Minor Appointments.

Fulham Infirmary, Hammersmith.—Miss Mary Isabel Jones has been appointed Charge Nurse at this infirmary. Miss Jones trained for three years at the Victoria Hospital, Burnley, afterwards working at a surgical home at Bristol.

Walsall New Workhouse Infirmary.—Miss Marion A. Foggett has been chosen to fill the post of Head Nurse at this new infirmary. Miss Foggett received her training at the Birmingham Workhouse Infirmary, where she was afterwards night superintendent.

Derbyshire Royal Infirmary.—Nurse N. Stanley and Nurse N. Oldacre have been appointed Staff Nurses at this hospital. The former received her training at the Royal South Hants Infirmary, Southampton, the latter at Addenbrooke's Hospital, Cambridge.

### Our American Letter.

The Brooklyn Nurses' Club last year appointed a committee to inquire into the question of establishing some form of general nursing organisation, but the plan was finally abandoned. The nurses have, however, become convinced that some steps must be taken on the subject of remuneration and of how to provide proper nursing for people unable to pay for trained nurses, both for their own protection and for the benefit of the public. A large meeting was accordingly held last month at the Hoagland Laboratory, the chair being taken by Mrs. Truman J. Backus. Dr. Florence Leigh-Jones discussed the question of organisation from the nurses' point of view, and a paper was read by Dr. Jennie V. H. Baker on "The Necessity of Immediate Organisation." Some practical suggestions were made as to the formation of a society, and Miss Isabelle Merritt, superintendent of the Brooklyn Training School, was elected president.

The Training School for Nurses, Western Pennsylvania Hospital, has considerably increased the number of its male pupils during the past year. Beginning with two, there are now twelve in training, and so far the experiment has proved successful. The male pupils attend the lectures given by the medical staff, and receive the same class instruction with the other nurses, their work in the wards being something beyond that of untrained orderlies. There have been several demands for male nurses for private work.

St. John's Hospital, Lowell, Mass., will sadly miss Sister Beatrice, who has for twenty-two years laboured in its service, and under whose administration it has grown and improved. Lately near New Orleans an institution for the care of lepers has been established, and it proved not easy to find nurses for the work. Sisters of charity finally offered themselves, amongst them Sister Beatrice. Very warm and affectionate wishes will follow her in her new labour of love.

By the will of the late A. W. Stearns, of Lawrence, Massachusetts, a bequest of 50,000 dols. is left to the General Hospital, to be made over in eight years' time. There is a condition attached to the legacy, that the name of the institution shall be changed to the "Artemus W. Stearns' Hospital." Should this be objected to the money will be diverted into other charitable channels.

The New York Training School for Nurses recently held its annual reception, and a very pleasant gathering was the result. The rooms of the association were charmingly decorated with flowers, and some two hundred and fifty guests accepted the invitation to be present.

### Everybody's Opinion.

[Correspondence on all subjects is invited, but we cannot in any way be responsible for the opinions expressed by our correspondents. No communications can be entertained if the name and address of the correspondent is not given, or unless one side of the paper only be written on.]

### HOLIDAY HOMES.

"A Member of the R.N.P.F." writes: Being a constant reader of The Hospital, I see how often you are bothered by nurses asking about holiday homes, &c. The Y.W.C.A. has seaside and country homes of rest, also holiday homes, all over Great Britain, of which a list can be obtained from the "Secretary, 316, Regent Street, W.," for three-halfpence, post free. Two summers ago I spent my holidays at one of them (it was "Manston House," Largs, on the Clyde), and I had a delightful holiday. The matron was so good to us all. The board and lodgings in the home amounted to about 10s. 6d. a week. I think a few of our London nurses might be glad to know of a home at such a reasonable cost; it is a lovely spot, facing the sea, with daily sailings on the Clyde, up the Kyles of Bute, &c. One more place let me mention, that is at Portrush, North of Ireland. The terms are a little higher there, 12s. 6d. per week; it is very bracing, and a lovely spot, and the superintendent is a nurse. I have not been there myself, but intend spending my holidays there in August; the name of the house is "Craig Dhue." Nurses who are seeking such places at a reasonable cost could not do better than consult the pamphlet I have mentioned. It is not necessary to be a member of the Y.W.C.A. I see a nurse is asking about the Channel Isles. There is a Home of Rest at "The Vale," Guernsey, terms from 7s. 6d. upwards.

### THE TRUTH OF THE MATTER—THREE YEARS' TRAINING FOR NURSES.

"A Provincial Matron" writes: I quite agree with "London Matron" that a probationer owes some service to the hospital where she has received her training, at the same time I do not think that three years' training is an "absolute necessity," especially in the case of those nurses who are being trained for "private nursing institutions." For the latter I would suggest that at least one out of the three years should be spent in a good "fever training school," of which, fortunately, there are many. Having, during my nursing career, been attached to a "private nursing institution," I know, from my own experience, that more than half a private nurse's cases are "fever," which, as we all know, requires most skilful nursing. And yet, how often is a private nurse sent to a fever case without the slightest knowledge of nursing it. In the paragraph on "The Truth of the Matter" in The Hospital for May 30th it is said that practical medical men and nurses consider that two years' training is sufficient for either district or private nursing. I think not many old nurses will agree to this. I for one certainly do not; in my opinion, for private and district work, thoroughly trained skilful nurses are required; and I am sure that a thorough, good, all-round training cannot be gained in two years. I am sincerely glad to see that the committee of the Bristol "Nurses' Training" Institution are being urged to take a step in the right direction.

### Notes and Queries.

### Queries.

(79) *Holland Institute.*—Will you kindly tell me if the directors of the Holland Institute have advertised for nurses lately?—*M. J.*

(80) *European Hospital Abroad.*—Can you tell me the names of European Hospitals abroad? I have been nursing in India, taking charge of male wards in a general hospital, and wish to find similar work somewhere abroad. Is there a hospital at Gibraltar?—*F. C.*

(81) *Emigration.*—An intending emigrant would be glad to know what prospects a trained nurse would have in South Africa? Would it be safe to depend on getting private work?—*Buxton.*

(82) *Holidays.*—Could a nurse holding a sister's post in a large general hospital justly claim a month's holiday money in lieu of her month's holiday under the following circumstances? She has no desire to remain in the service of the hospital longer than a twelvemonth, eleven months of which have already passed. The rules plainly state that a month's holiday is given to sisters annually, and she thinks it will be her turn to go next. She thinks it would be more honourable to tell the authorities her intentions rather than to take her holiday in the usual course, only to return to give a month's notice. At the same time she does not wish to lose her month's holiday or its equivalent, a month's money, which would enable her to take a little leisure whilst seeking another post.—*Madras.*

### Answers.

(79) *Holland Institute* (*M. J.*).—Not in our columns.

(80) *European Hospital Abroad* (*F. C.*).—You will find all such hospitals given in Burdett's "Hospitals and Charities," (Scientific Press, 428, Strand). Write to the institutions. There is the Colonial Hospital at Gibraltar, under the control of the Colonial Government, which is nursed by Englishwomen, but vacancies are filled from the London Hospital through the Government.

(81) *Emigration* (*Buxton*).—We are always cautioning nurses against "depending" on getting private work in the colonies or anywhere abroad. Unless you have reliable private information about any place, or a definite engagement and prospect of work, and enough money to be able to wait for employment, it is most unwise to make any such venture.

(82) *Holidays* (*Madras*).—We do not think you could claim a month's pay in lieu of the month's holiday to which in any case you are entitled after a year's work, neither does it seem that you are in any way constrained to state your intention of leaving before going for your holiday. We understand from your letter that you are prepared, on returning, to remain for the month after giving notice, and would doubtless be willing to meet the wishes of the authorities as to the exact date of leaving. You could write while away and give notice of your intention so as to give the matron a little more time to fill your place.

### For Reading to the Sick.

HOLINESS.

**Motto.**

" 'Tis only noble to be good."

**Verses.**

We cannot reach our Saviour's purity,
Yet are we bid, " Be holy e'en as He ! "
In both let's do our best !—*Herbert.*

Are we not holy ? Do not start !
It is God's sacred will
To call us Temples set apart
His Holy Ghost may fill.—*Adelaide Proctor.*

" Religion " never did betray
The heart that loved her ; 'tis her privilege
Through all the years of this our life, to lead
From joy to joy ; for she can so inform
The mind that is within us, so impress
With quietness and beauty, and so feed
With lofty thoughts, that neither evil tongues,
Rash judgments, nor the sneers of selfish men,
Shall e'er prevail against us, or disturb
Our cheerful faith, that all which we behold
Is full of blessings.—*Wordsworth.*

Lord, let my guardian angel school
My steps, severely kind ;
And let the march of ages rule
The motions of my mind.—*John Charles Earle.*

With Jesus for our guide,
The path is safe, though rough ;
The promise says, " I will provide,"
And faith replies, " Enough."—*John Newton.*

Simple rule, and safest guiding,
Inward peace, and inward light !
Star upon our path abiding,
Trust in God and do the right.
—*Norman McLeod.*

**Reading.**

Bought with the precious blood of Christ ? It is this which makes our peace sure ; our holiness sure ; our eternity sure !—*Herman Douglas.*

Go forth in the strength of the Lord, and expect to do great things, and also to prevail.—*Newton.*

Almighty God, the fountain of Holiness, Who by Thy Word and Thy Spirit dost conduct all Thy servants in the ways of peace and sanctity ; grant unto me so truly to repent of my sins, so carefully to reform my errors, so diligently to watch over all my actions, that I may never willingly transgress Thy holy laws ; but that it may be the work of my life to obey Thee ; the joy of my soul to please Thee ; the satisfaction of all my hopes, and the perfection of my desires, to be with Thee in Thy kingdom of grace and glory.—*Cloud of Witnesses.*

This is the great foundation of a true and holy life. Our hearts must yearn after Him ; must pray that we may be with Him ; must fear to be parted from Him ; must long to live in His presence—finding it shelter, and safety, and peace ; and then He will manifest Himself unto us. Even here, whilst He seems for a season to hold us from Himself, and not to suffer us to come into the ship with Him ; whilst He sends us back to life, to its business, its cares, its pleasures, and its sorrows, and bids us enter into them heartily ; even here, He will be with us ; in, and through all these acts of service, He will be at our side, and His presence will give truth, and reality, and safety to our lives.—*Bishop Wilberforce.*

### Wants and Workers.

[THE attention of correspondents is directed to the fact that " Helps in Sickness and to Health " (Scientific Press, 428, Strand) will enable them promptly to find the most suitable accommodation for difficult or special cases.]

HOLIDAYS AND HEALTH.—Nurse Parry, district nurse, Ruthin, North Wales, will be glad to give nurses who are thinking of holiday arrangements information as to lodgings in a beautiful part of this mountain country.

### Sketches of Hospital Life.

SOME of the living pictures which the wards and out-patient departments of a great hospital supply in such prodigal profusion might usefully be produced in these columns as affording food for thought, and opportunity for reform and succour. The inner life of the poorer residents of great cities nowhere finds more pathetic revelation than within the walls of a hospital. There are innumerable cases which illustrate precisely how and where our social system fails to reach and to aid some of the most deserving amongst the poor. Child succour, moral improvement, the elevation of character, the protection of the weak, and the building up of the unstable are branches of work which might be helpfully carried out by intelligent and zealous philanthropists if they would consent to place themselves in communication with the managers of our hospitals, or by qualifying as governors tender their services as permanent visitors with the view of pursuing the practical aspects which hospital life in its wonderful variety never fails to present year by year. We have given some thought to this subject, and by way of illustration we propose from time to time to publish sketches of hospital life, providing our correspondents will supply us with the necessary material, of which there is abundance. The following sketch has been sent to us from New York. We shall publish another similar sketch, which is also taken from an American hospital, and shall hope to receive similar incidents from workers in British hospitals at home and in the Colonies.

### IN A PROVINCIAL HOSPITAL.

He was a small grimy boy of perhaps six years, attired in " looped and windowed raggedness," whose home was apparently the streets. An ice-waggon had passed over his leg, breaking it in two places. After a few weeks in the hospital, when the accumulated dirt of years had been washed away, and good food had filled out the wizened face—he was changed from an impish boy into a pretty lovable child, whom any mother would have been proud of. But no mother called to claim Peter. He soon won his way into the hearts of the nurses and visitors, especially as this was not a children's hospital, toys and picture books poured in, he was taught to read, and also not to swear.

Lying in bed for months, he never seemed to know a moment's weariness, his favourite amusement being reading or reciting in a sweet little voice, for the amusement, or boredom at times, of other patients. At last he was strong and well, no excuse for keeping him any longer. Our adopted child must go. An unprepossessing individual (all poor Peter seemed to have in the way of relatives) came for him, " as he was now at work he could take better care of him." With many misgivings, we said good-bye, and Peter departed, leaving no resemblance to the vagabond brought to us in the ambulance, with round rosy face and new suit, the many pockets filled with treasures, and with faithful promises of coming to see us. Months passed away ; in the many changes of hospital life the small patient was almost forgotten. When, one day, a forlorn, ragged urchin turned up he seemed unknown to us ; but it was Peter, relapsed into barbarism and filth. His brother, he informed us, had been sent to jail for stealing a watch.

He stood uneasily in the corridor, like an animal at bay, ready to take flight at a moment's notice, but evidently throwing himself on our mercy, the only friends, perhaps, he had known in his few and evil days. We managed to keep him for a few days till removed to a home. But our Peter was gone ; the flower of childhood was dead ; it seemed impossible it would ever be revived.

Poor little waif, whom " the strange hurly-burly of the thing called life " had defrauded of everything.

" Still all day the iron wheels go onward,
Grinding life down from its mark,
And the children's souls, which God is calling sunward,
Spin on blindly in the dark."

## Figures and Tables

**Fig. 23. f1:**
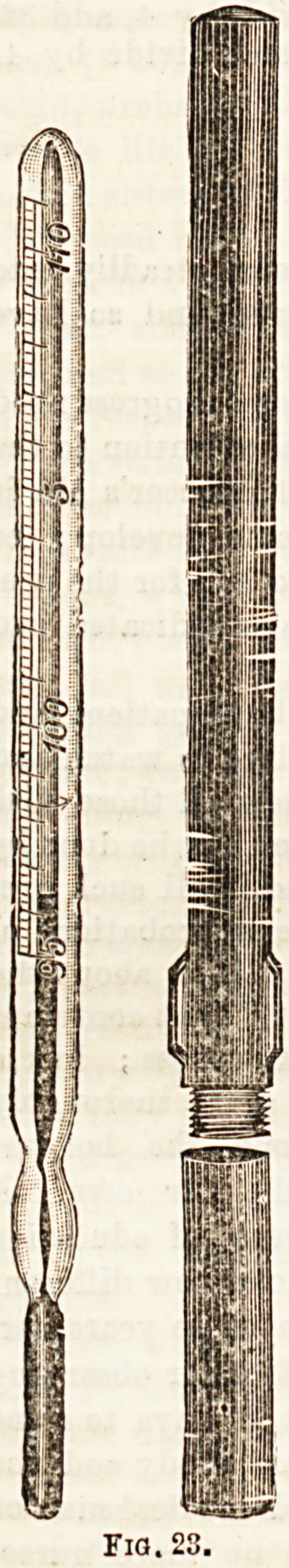


**Fig. 24. f2:**